# “You Don’t Know Me so Don’t Try to Judge Me”: Gender and Identity Performance on Social Media Among Young Indian Users

**DOI:** 10.3389/fpsyg.2022.855947

**Published:** 2022-06-17

**Authors:** Sramana Majumdar, Maanya Tewatia, Devika Jamkhedkar, Khushi Bhatia

**Affiliations:** Department of Psychology, Ashoka University, Sonepat, India

**Keywords:** social media, social identity, SIDE, CMC, gender, feminism

## Abstract

Social media is the preferred communication platform for today’s youth, yet little is known of how online intergender communication is shaped by social identity norms. Drawing from the Social Identity and Deindividuation Effects (SIDE) approach, we argue that through depersonalization, online interactions are marked by the salience of social identities and identity performance conforming to perceived norms of behavior (traditional as well as developing). We specifically look at discursive terms and their meaning-making as a strategic performance of gender in uncontrolled social media interactions. We examined a corpus of 442 comments from selected public Indian Facebook pages in two phases over a span of 1 year (2020–2021). Thematic discourse analysis revealed established (#mansplaining, pseudofeminism) and emerging (choice feminism, MGTOW, #fuckboi etc.) discursive strategies within the major themes on feminism and antifeminism, men’s rights, intersectional feminism, and sexual behavior. These meaningful terms are used to modulate identity performance in a heavily contested space, reflecting both consolidation as well as mobilization functions, as proposed by SIDE. The findings highlight that intergender communication on social media is both dependent on existing offline norms, while challenging the same to create new discourses of gender.

## Introduction

There is considerable social psychological work examining interpersonal and intergroup interactions and its consequences. Yet, the majority of this is concentrated on face-to-face interactions. Today, a large part of our interactions are online and social media has become the preferred medium for this globally (Johnson and Callahan, 2015; Bulut and Kesgin, 2016). Reports suggest that young adults are communicating and building relationships online, more than *via* face-to-face communication (Velten and Arif, 2016). Consequently, interactions on social media are now a significant source of contact as well as conflict.

While interacting on social media, users not only meet as individuals, but also as representatives of their larger social identities (Hogg et al., 2004). The absence of face-to-face (FTF) interactions in online spaces can facilitate anonymity (Ehrlich and Stoerger, 2014), but chosen identity markers reveal one’s social identity, which allows them to be perceived in accordance with offline social stereotypes and prejudices (Cirucci, 2017). Gender is a social structure and group-based identity that determines social relationships and behaviors at various levels of the social world (Goffman, 1976; Armentor-Cota, 2011). When such a social structure permeates into the online setting, gendered communication norms are formed (Armentor-Cota, 2011; Rose et al., 2012; Spears, 2017). In the absence of personal identity markers and the presence of gendered norms of communication, gender identity becomes salient in online interactions (Armentor-Cota, 2011; Rose et al., 2012; Spears, 2017). This creates a “continuous communication loop” where gender identities shape interactions online, which in turn create opportunities and norms that dictate gender relations and expectations of behavior (Rose et al., 2012).

This paper aims to examine cross-gender communication, which is the “communication about and between men and women” (Ray and Pani, 2019) on social media, drawing from the Social Identity and Deindividuation Effects (SIDE) approach (Spears, 2017). Specifically, we take a look at the dominant discourses on gender that are popular online and become persistent references in communication. We examine written text as indicators of identity performance which reinforce and reconstruct online gendered communication. Through this analysis we hope to present a case for how gender interactions on social media are symbolic of social identity representations that are shaping gender interactions and discourses in the virtual space. Here, we acknowledge the presence of multiple gender identities which is beyond the scope of this paper. We analyze traditional cross-gender communication between men and women only, given the larger presence of the same and the novel lens of analysis that we are using with respect to social media use in the Indian context.

### Online Interactions and Gender

The focus on contact *via* computer mediated communication (CMC) has expanded over the last 2 decades. While initial research highlighted the equalizing nature of online spaces, others underscored the rising polarization on social media platforms (Amichai-Hamburger and Hasler, 2013; Cirucci, 2017). Work on gender and CMC reveals a similar 2-fold trend, where on one hand, the democratic nature of the virtual space is upheld for equalizing gender interactions and opening a space that disrupts established norms (Webb and Temple, 2015); there are significant gender differences in access to virtual spaces, and communication patterns online (Yates, 1997). Status and visibility differences between men and women can be seen on social media platforms like Twitter where individuals with disadvantaged intersectional identities, like women of color, receive less attention than white men (Messias et al., 2017). Referred to as the “Gender digital divide, the unequal access to, use and awareness of digital spaces” is a worldwide phenomenon, being particularly salient in the Global South (Antonio and Tuffley, 2014; Alozie and Akpan-Obong, 2016; Fatehkia et al., 2018; Joshi et al., 2020). In India, 67% men are Internet users, compared to only 33% women, with even fewer numbers in rural areas (Kala, 2019). Unlike Western social media usage which is becoming increasingly gender-equal (Greenwood et al., 2016; Tankovska, 2021), 78% Indian social media users are men.

There is also a complex manifestation of sexual behavior and norms on social media, where along with constructive experiences of gender construction, there are undesirable consequences of body shaming and exposure to sexual content (Davis, 2018). Research also suggests significant differences in how men and women present themselves as well as interact online (Hudson and Gore, 2017). According to Kivran-Swaine et al. (2012), men initiate more cross-gender friendships while women tend to express more positive emotions and use profile pictures more often. Analysis of Facebook profile pictures revealed that gender stereotypical traits that are dominant offline are represented online with pictures of men being rated higher on traits like active, dominant, and independent while women scoring higher on attractiveness and dependence (Rose et al., 2012). Armentor-Cota (2011) notes that while gender swapping and gender fluidity are often present; stereotypes exist widely and guide online communication to a large extent. For example, in how men and women resist or defend themselves online, distinct patterns emerged where men typically dominated and asserted their viewpoints as opposed to women who often justified or defended theirs. Cirucci (2017) found that women were more conscious and anxious about their posts and comments on social media sites. Through experimental findings, Spears et al. (2014) showed that men tended to dominate most online discussions where gender was salient.

Gender identity becomes particularly salient in online collective action for issues pertaining to gender itself, such as spreading awareness about feminism. With the advent of the fourth wave of feminism, there has been a growth in cyberfeminism on digital platforms where participants not only consume information but also actively participate in the movement through engagement (Jain, 2020). Language plays an important role in digital collective action, especially with the use of hashtags, which are effective tools to mobilize people for social change, raise awareness about important issues, and develop a sense of community (Storer and Rodriguez, 2020). The study of Yoder et al. (2010) on self-labeling found that self–categorization as a feminist predicts engagement in collective action online. Moreover, engaging in Twitter activism in response to sexism was found to promote an enactment of women’s social identity, which led to further mobilization for collective action (Foster et al., 2020). Discussing the “Gender digital divide” in developing countries, Antonio and Tuffley (2014) note that one of the most significant benefits of the internet for women is the potential for forming social networks, self-expression, and a collective identity formation.

However, irrespective of the definitive work on gender and online interactions, there is limited literature examining cross-gender interactions from a social identity and intergroup relations perspective. We were interested in locating gender as a salient social identity category and exploring the influence of norms in shaping communication on social media. To do this, we borrowed from the Social Identity model of Deindividuation Effects or SIDE framework which is useful in contextualizing and explaining CMC. This approach, rooted in social identity and social categorization theories is particularly suited to explore social media interactions among members of historically contested groups, and examine how group identification and the presence of norms facilitate identity performance, assertion, and opposition to outgroups (Reicher et al., 1995; Perfumi, 2020). Thus we combined parallel but seldom overlapping approaches by examining online gender performance and its various strategies (underexplored in psychological literature beyond interpersonal approaches) from the lens of contested social identities, existing and emerging intergroup relations (Webb and Temple, 2015).

### SIDE and Identity Performance: Theoretical Framework

According to social identity theory, interactions between individuals can be located on a continuum between social identity salience, to the dominance of individual identity where interactions are interpersonal and directed by personal motives and desires. Applying this to CMC, Reicher et al. (1995) propose that in visually anonymous communication, the invisibility of personal identity leads to the salience of social identities, resulting in behavior that is in-group normative, through the process of depersonalization. Individuals self-categorize and perform their social identities in ways that are perceived to be normative, as well as evaluate others in comparison to the prototypical members of the outgroup (Lea et al., 2001; Postmes et al., 2001). Interactions on the internet are marked by social identity cues and narratives that are dominant and guide these conversations (Rains et al., 2017). These dominant discourses also influence how identities are performed online.

Identity performance is the “purposeful expression (or suppression) of behaviors relevant to those norms conventionally associated with a salient social identity” (Klein et al., 2007). This performance goes beyond self-presentation as it is motivated by concerns for social identity. According to the “strategic aspect of SIDE,” this serves both functions of identity consolidation (protecting, upholding, and defending the salient social identity) as well as identity mobilization (acting in pursuit of group goals which, for instance includes, antagonizing the outgroup to prove their illegitimacy). Mobilization is particularly important as it closely relates to collective action and how social categories can shape norms, expectations, and social realities. Thus, in interactions between men and women when gender is salient as a social identity, the communication is not only shaped by the awareness of this identity and its normative performance, but also driven by the need to uphold the in-group identity, defend against the “other” as well create opportunities and narratives that the group can strive toward. Online, identity mobilization includes discursive strategies that establish group norms, underscore resistance, and often result in outgroup denigration (Rains et al., 2017). Such interactions can create new rules of communication, new social realities of gender which move beyond online interactions to become larger gender discourses.

Research following the SIDE approach has revealed interesting processes that support its theoretical claims. For instance, Spears et al. (2002) showed that perceived social support in online interactions can facilitate collective in-group action and resistance to powerful outgroups. Rains et al. (2017) found that outgroup presence, previous hostility toward ingroup and intergroup bias were important predictors of online incivility. Applying the SIDE model specifically to the analysis of gender, Spears et al. (2014), confirmed how women and men managed their identities differently in gender salient online communication. However, most of these were lab based experimental studies that do not necessarily address how identity performance and its various strategies are employed in uncontrolled social media interactions. We expanded this lens to look at how individuals use discursive techniques as strategies to perform their identities. Klein et al. (2007) emphasize the importance of discursive strategies in creating, maintaining, consolidating, and mobilizing social identities in its performative function. Examining intergroup relations through the lens of discursive techniques aid in understanding how identity-based norms prevail and shape interactions and are co-constructed through these very social interactions (Durrheim et al., 2015).

In India, as social media use has expanded, so have conversations on gender norms and relations. More recently, social media have become increasingly politically polarized (Neyazi, 2017) and witnessed intense debates and discussions around themes of sexual harassment (Pain, 2020). Going online and participating on social media is often marked by anxiety and apprehension for Indian women. Women’s online experiences can be unpleasant, with repeated encounters of sexually inappropriate or aggressive behavior (Karusala et al., 2019). Yet, the presence of women on social media has been viewed positively by many as a forum for feminist activism. Many women have participated in online campaigns on women’s safety, harassment, menstruation, and hygiene (Mirani et al., 2014) and are using platforms like Twitter to actively engage in conversations around gender-based violence (Gurman et al., 2018). Social media becoming a significant space for gender performance, resistance, and reconstruction are a globally relevant phenomenon. Ogan and Baş (2020) showed how social media were used as a platform for solidarity, resistance, and emotional expression toward violence against women in Turkey, Sylwander and Gottzén (2020) study revealed the strategic implications and resistance to gendered terms in online communication in Sweden, and, Cook and Hasmath (2014) presented a cross-cultural analysis of participation in the online #Slutwalk campaign, indicating several discourses around feminism, intersectionality, and the construction of gender. Thus, examining social media discourses on gender is not only widely applicable but also presents relevant contemporary debates that will help shape shifting gender understandings. For example, discussing the Men’s Rights Movement in India, Basu (2016) points out that the MRM and similar arguments from men often get represented in a typical anti-feminist discourse that is met with immediate retaliation or dismissal which can neglect underlying anxieties. The author notes that changing gender norms, resistance and laws are deeply embedded in a historical system of patriarchy that has consequences for men and women, and questions around contested feminism in a post-colonial society. This study adds to the relatively limited work on CMC and gender in the Global South (Nova et al., 2019), diversifying this research, adding to the SIDE/CMC literature and its application in varied contexts and through multiple methods.

### The Present Study

**Figure 1:**
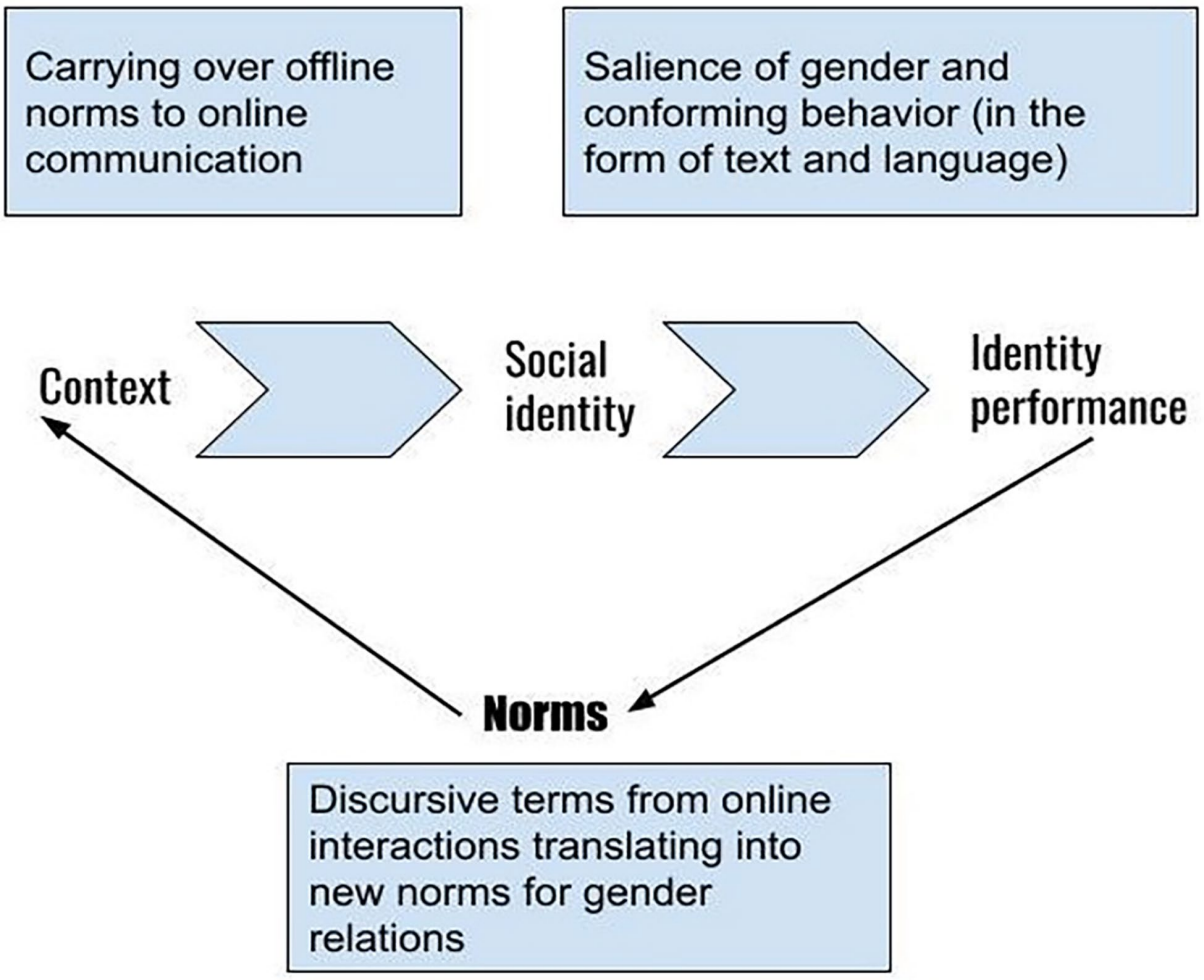
Offline norms to online identity performance.

We explore online intergender communication among Indian social media users through a discursive lens. We approach this analysis from a social identity perspective rooted in social psychological theorization. We argue that in these discursive strategies, users actively perform their identities by reiterating existing and emerging gender norms that shape gender activism, resistance, and anxieties in online spaces. The analysis is informed by a three-step method (i) the context (existing gender norms in India), (ii) social identity (gender as the salient social identity), and (iii) identity performance (as proposed by the SIDE approach). The intersection of these three leads to the emergence of new norms of intergender communication, marked heavily by the use of meaningful terms and language, reshaping the larger context of gender relations (Figure 1).

“Discourses are conversations or talk with an agenda” that represent and govern the present nature of social relationships and how individuals make sense of them (Singer and Hunter, 1999). Rooting itself in discursive psychology, discourse analysis assists in providing an understanding of how social identity is constructed, as well as the effects of such identity construction (Ainsworth and Hardy, 2004). Discursive studies of identity thus challenge many of the traditional assumptions of psychological research by showing how social resources construct individual identity (Potter and Wetherell, 1987). Hence our aim was 3-fold (i) to identify and examine consistent language patterns in intergender communication on social media, (ii) to extract meaningful emerging discourses representing gender norms, and (iii) to analyze these themes according to the presented theoretical design: history of gender relations, salience of gender as a social identity category, use of gender normative language as performance of the salient identity, and the emergence of new norms for communication and behavior.

## Materials and Methods

### Selection Procedure

To explore discursive themes prominent in intergender social media interactions, we started out by scoping different social media forums to get a preliminary idea of the conversations. We chose to include comment threads on public pages on Facebook as our main data corpus. Webb and Temple (2015) identified Facebook as one of the leading online forums where gender performance can be studied given the presence of profile pictures, description and the interactive aspect of responding to posts and comments. Facebook is also the most used social media platform in India (except YouTube), which has public pages on a wide range of topics with a diverse socio-demographic participation (Chakravarti, 2021; Kemp, 2021). On Facebook, we were interested in looking at pages which included those where gender was explicitly relevant (ex: pages on feminism) as well as others which were more generic and news oriented (ex: political and entertainment news). This was done to map the landscape of gender discourses across a range of pages with the intent of understanding if the specific terms were only used in gender-polarized pages or regardless of the content of these pages. We selected three public open Indian Facebook pages that emphasized interactions around gender and gendered behavior—*Feminism in India*, *She The People*, and *Journal of an Indian Feminist*; and one page that showcases interactions between individuals of diverse socio-political views, *The Print*. Our data corpus timeline was particularly aimed at capturing the significant role that social media has played over the last year (2020–2021), in light of the COVID-19 pandemic and the resulting lockdown. While there was a global increase in social media use, the experience of the same was mixed (Coronavirus: 87% Increase, 2020; The two sides of social media during COVID-19, 2021). Specifically in the Indian context, conversations around online violence, gender trolling, and sexual harassment almost tripled during the COVID-19 period, with gender trolling having the largest share of 47% of such conversations (Quilt AI and ICRW, 2021).

A purposive sampling was done for posts in a two-phased manner—once in February–March 2020 and again during the same time in 2021—where we looked for words and expressions that were repeated and used as hashtags or specific meaningful terms (Libutti, 1999). The criteria for selection of the comments were such that we only used the “top” comments on each post that had Facebook users replying to each other. One can choose the order in which comments are displayed on Facebook; we used the “Top Comments” order, which means that the comments with the most “likes” and “replies” were displayed first (Mavoa et al., 2017). When comments were reported, usernames were removed, but the comments were copied unedited; as a result, any spelling errors, grammatical mistakes, spacing errors, or other typographic errors were reproduced to present the posts as precisely as possible (Rademacher, 2018). We used the manual extraction (copying and pasting data into a spreadsheet) method to collect our data (Abramson et al., 2014; Franz et al., 2019). In the first phase of data collection in 2020, 23 comment threads with a total of 72 comments from gender relevant pages and 14 threads of 37 comments from *The Print* were included in the sample. For the second phase, the sample consisted of 41 comment threads with a total of 110 comments, and 68 comment threads and a total 223 comments from *The Print*. In total, 442 comments (*n* = 442) were looked at four levels, as indicated in Table 1.

**Table 1:** Levels of a comment.

Comment levels	
Text	The content of a comment
Hashtags	The hashtags (if any) used in the comment
Terms	Gender-specific terms used in the comment
Interaction	Presence of inter-gender interaction

### Data Analysis

The collected data were analyzed using thematic discourse analysis. The combination of thematic analysis with discourse analysis has been used previously (Taylor and Ussher, 2001; Clarke, 2005) and is specifically recommended for analysis of internet-based discussions forums (Simoni et al., 2014; Botelle and Willott, 2020). This method identifies themes in a text within a constructionist framework, focusing both on the rhetorical design and on the ideological implications of the themes (Clarke, 2005). As part of social discursive psychology (Harré and Gillet, 1994; Edwards and Potter, 2005), the relevance of symbolic artifacts in a community (languages, rituals, and relations) is emphasized to better understand their cultures. These social and discursive dynamics are important in virtual communities as well as individual user profiles who express emotions, beliefs, and desires through their discursive engagement online (Scardigno and Mininni, 2020).

Subject matter, word function, and discursive characteristic were used to assign codes at the sentence or lexical item level (Mavoa et al., 2017). The code frame was built through a mixture of deductive and inductive coding. The deductive code development was partly adapted from the approach of Jones et al. (2019) to studying misogynistic online harassment. An inductive reading of the comments found that this failed to capture some of the forms of interactions that were present and thus further categories were added. The code frame was then refined inductively, drawing on observations and analysis from the close reading of comment threads. This combination of deductive and inductive coding provides a more comprehensive code frame that captures diverse forms of interactions. Such an approach was supported by Freelon (2013, p. 1186) who states that “researchers should feel free to appropriate and/or develop additional conversational measures” and “it may not always be necessary to measure all features as some will almost never be present in certain forums.” An inter-coder reliability test was conducted with two trained coders on 10% of the sample (*n* = 44), using Krippendorff’s alpha; the reliability scores were 0.944 and 0.956.

We followed six step guide of Braun and Clarke (2006) to analyze the data. The research team consisted of three researchers working on the data simultaneously. First, we read through the data set several times to familiarize ourselves with the material and had discussions to note initial ideas. Repetitive, relevant and meaningful discursive terms were identified as initial codes. These were then grouped together and categorized to form coherent themes that represented a larger discourse. The interpretation of these themes was done by reading and re-reading the text, discussions among the researchers and reference to relevant literature. This was overall informed by the theoretical approach by considering relevant concepts at every step of the analysis (Taylor and Ussher, 2001; Braun and Clarke, 2006). As we collected data in two phases, we also reviewed the two datasets together to compare and define overarching themes. There was significant overlap and similarity between the codes generated from Phase I (2020) and Phase II (2021). Hence, we decided to combine the codes from both phases to form themes that represent discourses across the span of this one-year period, as shown in Table 2.

**Table 2:** Themes and codes overview.

Theme	Code	Frequency
Feminism and antifeminism	Feminazi	27
	Pseudo-feminism	21
	Whataboutery	17
	Victim card	19
	Feminists should avoid marriage	15
	Gold Digger	12
	Motherhood seen as epitome of womanhood	11
	Feminists seen as selfish women	9
	Feminist Fascists	8
The Manosphere	Misogynist	35
	MGTOW	19
	Incel	19
	Male Bashing	17
	Mansplaining	17
	MCP	11
Intersectional identities	Urban Feminist	14
	Choice feminism	8
	Savarna Feminist	7
	Dalit Feminism	6
Threat, sex and violence	Slut	23
	Fuckboi	19
	Simp	18
	Small Cock	8
	Sissy	8
	Whore	7

### Researcher’s Position and Ethical Consideration

We would like to acknowledge our positions as female researchers and social media users, and the influence of our gender identity and personal experiences on the research. However, we upheld the importance of a non-evaluative and non-judgmental stance, engaged reflexively with our social positions, and proceeded with utmost rigor at every step of this study. We had a team of multiple researchers and had regular discussions with a larger group of male and female researchers for feedback on our analysis. This process facilitated a reflexive journey rooted in collaboration and collective critical consciousness (Mao et al., 2016). The study was approved as part of a larger project on online contact and intergroup attitudes by the Institutional Review Board of Ashoka University, India. We maintained complete data secrecy and confidentiality by anonymizing names and any potential identifiers. Facebook allows the use of data from public pages and since Facebook comments are publicly accessible, no consent was necessary or requested from either the page owners or users to evaluate the comments on their posts (Abramson et al., 2014). Therefore, we only included posts and comments from public open pages for our analysis.

## Discussion

After coding and organizing the data, four major themes emerged that have been discussed below. Within each we refer to language and terms that signify what these discourses represent and the new identity norms they facilitate in the process of interaction. The discussion is also supported by direct comments from participants presented in the following section.

### Feminism and Antifeminism

One of the most common discourses of contestation was the idea of feminism—what it means and how it is practiced. Firstly, in line with social identity theory, the notable effort to create an exclusive and distinct in-group (feminist) and outgroup (antifeminist) was prominent (Durrheim et al., 2016). This was used both for the purpose of mobilization as well as consolidation, wherein group members willingly reiterated terms and meanings to ensure that their own identity is accepted as a valid ingroup member (Klein et al., 2007). Members asserted and clarified their understanding of the feminist ideology by emphasizing that women should have their individual freedom, wear clothes of their choice, not face any societal pressure, or that feminism did not equate to “men haters” as seen in comments like: “It’s completely their own choice and no one can dictate a women what she should do!”; “If there were one article that ought to have convinced men that feminists aren’t out to get them, this should be it.” There were many instances of asserting the role of *feminism against patriarchy*, reflected in this comment:

Patriarchy is not gone, gender equality is still an aspiration for most societies, so yes feminism is still fighting for freedoms and will continue to, whether male entitlement likes it or not. And no men will not tell us how to fight and which brand of feminism they like or prefer!

The larger antifeminist discourse in our data included three consistent patterns of thought—the first was the perceived lack of “feminists” to accept critique, second was the perception of feminists as hyper aggressive and reactionary; “Many of these feminist on this page will react very violently and aggressively if something will not match with their views.” Lastly, there were assertions on female role stereotyping. The discursive connotations of female gender role stereotyping broadly reiterate that women should focus on their marriage, calling out female emancipation as being responsible for divorces, questioning their role as a mother and calling them a *“gold digger.”* In our data, telling women to keep their emotions in check, asking if they were menstruating or if it was their *time of the month* and blaming their hormones for their behavior was common. Jones et al. (2019) have reported such presence of gender stereotypes in Twitter comments, where sexism encompassed references to “get back to the kitchen” and “make me a sandwich.” One of the comments in our data also suggested something similar: “Apparently, a feminist has never experienced the joy of ironing a shirt and making a sandwich. No wonder they are chronically triggered.” We also found the stereotype of the *female emotional brain*, allegedly clouding women’s scope for logic. Women were often pronounced as too emotional to evaluate the status quo logically and rationally, to the point of being paralleled to children. Their inclination toward hyperemotionality was correlated to the lack of logic (Jones et al., 2019). Importantly what was observed repeatedly was the need to maintain and establish category distinctions with “you women or your kind.”

#### (Re) Defining the “Feminist”

Within the feminist/antifeminist discourse, we identified the emergence of newer terms that represent specific definitions of these ideologies, for instance, *Feminazi*. The term originated in the 90s, when Rush Limbaugh described it as “a feminist to whom the most important thing in life is ensuring that as many abortions as possible occur.” This discursive understanding of *feminazi* as an avoidance of motherhood and an act of “selfishness” was apparent in our data as participants posted comments like:

If women like you and the feminazis here are incompetent to be a mother, abstain from that. Motherhood is a selfless act and most selfish women nowadays do not want to leave their comfort for their kids. That is postpartum depression in the majority of cases. Pure selfishness.

Carrying a strong weight with the “nazi” suffix, today the word is rather casually used such that in our corpus it was the most frequently appearing. Comments like “Leave these feminazis. These vultures always have problems in everything” and “Being a feminazi it’s her birthright to be a hypocrite. So let her be” used the word almost as synonymous with radicalizing the “feminist” and “female.”

A second term was *Pseudofeminist*; which has been defined as a person who claims to be a feminist but ignores the main point of feminism, i.e., equality. This was often seen in the form of male and female participants questioning the feminists and “correcting” it as per their own ideology; one commented that “Most of the feminists do speak hate against men and then say they are just supporting women. So first decide the line of difference between Feminism and pseudo-feminism and then ask us to choose to be feminist or not.”

We found the use of anti-feminist terms by women as well. Here, women dissociated from the so-called feminists or used the same kind of retaliation as by non-feminists (usually men). Most such female participants questioned the idea of feminism: “Pseudo feminism is not feminism. Get your fckin facts straight today. And the women you talk about doing crimes on men are criminals. Start seeing beyond gender if you ever wanna mature.” Research has shown that women and groups of women who self-identify as “non-feminists” or “anti-feminist” often reiterate the discourses largely popularized by Men’s Rights groups (DeKeseredy et al., 2015). Some women feel alienated by the dominant feminist discourse, especially if they are not directly affected by the arguments that shape the normative standards of the ideology, such as equal rights in the workplace (Lorenzi-Cioldi, 1991). Identity performance in front of an outgroup can be both threatening and intimidating. While this leads to some members upholding their norms, being defensive and vindictive toward the outgroup, other individuals may refrain from identifying with the ingroup (Klein et al., 2007).

A third prominent theme here included an assertion that women and feminists accrue power and sympathy through the escalation of “false rape claims” or by “*playing the victim*.” This was visible in comments like: “Just another fragile feminist not happy she will not get away with self-victimization narrative!” *Playing the victim* has become an increasingly common discourse around women specific crimes in India in the recent past with many pointing out that the existing laws on domestic violence, rape and sexual assault are heavily biased toward women, who can easily manipulate and exploit the system at the cost of innocent men (Mishra, 2019; Navin and Jangid, n.d.). Within the discourse on feminism, “playing the victim card” has been used as strategies for counterattack, often by other women, since it positions those who voice their stories as weak (Donaghue, 2015). Issues of victimization brought up by women were met with a strong assertion of *whataboutery*, whereby members (mostly men) denied relevance of the female identity experience and rather questioned them about issues of ‘importance’. Comments like “what about the guy who committed suicide because of the extreme harassment by his wife?” directly raise the “what about” question, while others like “media is busy with its agenda of gender discrimination even during a pandemic” indirectly deny the relevance of gender.

Along with instances of women resisting perceived notions of feminism, as well as men aligning with the same, there was a notable amount of solidarity. Women spoke in support of women and men supported men. The solidarity was also expressed by shaming the other and their lack of knowledge, comprehension, and compassion. These were instances of discursive activism on contested and volatile themes seen in comments like, “Stop femsplaining misandrist. We do not need women to tell what should a man do or how should he express his emotions. We know what is best for us.” The scope of CMC to arouse collective emotions, perceptions of commonality, connection and disadvantage, and the ability to express opinions that are believed to be shared by the ingroup (Spears and Postmes, 2015) enhances in-group solidarity when categories are salient, and exchanges are particularly antagonistic.

### The Manosphere

Closely connected, but distinct from the discourse on feminism, was the discussion around men’s rights and their position in a transitioning society. This again involved two major threads—the first was a description of the “manosphere,” primarily by women, and the second was the assertion of Men’s rights, largely by men. The manosphere was referred to as a misogynistic space; an identity represented by *MCP (Male chauvinist Pigs)* with not only salient normative markers (misogynistic) but also associated with negative traits of being uneducated and violent; a few comments read:

“Why do not you go drink with your loser MCP buddies, cry and complain that women are not “traditional” like before, dare to talk back to men, dare to wear eyeliner, cry and wail about it, then go home and beat up your wife to you know, put her in her place? THAT will make you feel like a man.”

“The fact is the inbuilt misogyny of our society. But to see that, one needs to be well-read, cultured and have a balanced mind. Too much to expect from an MCP!”

In these exchanges, we see a denunciation of outgroup values and traits which is also rooted in context, wherein the speakers are challenging traditional societal (patriarchal and sexist) norms defining gender roles. Thus discursively, using terms that describe the “manosphere,” speakers are subverting established norms and performing their salient identities. This was often met by counter claims of Male *Bashing*, a term used to describe the unreasonable and unnecessary disregarding of men, complimenting the previously mentioned “hyper feminist” discourse, as seen in comments like—“Every toxic feminist on this page are just bashing men aggressively. It just shows huge double standards of these feminazis”; “Typical men hating bigot feminist playing victim card and bashing men. What u are doing to me is just mental harassment if a man say the same thing to u.”

The internet has been key to the popularization of men’s rights activism and discourse (Lily, 2016; Schmitz and Kazyak, 2016). While the manosphere includes a variety of groups, including Men’s Rights Activisms (MRAs), men going their own way (MGOW), incels (involuntary celibates), and so on, they share a central belief that feminine values dominate society, which is a fact suppressed by feminists and men must fight back against an overreaching, *misandrist* culture to protect their very existence (Marwick and Lewis, 2017).

*Men Going Their Own Way* refers to the group of men who have vowed to not pursue romantic relationships with women to focus on their self-development and preservation (Jones et al., 2019). Comments like “Feminism is a disease, MGTOW is the cure” highlight how the discursive understanding of MGTOW is rooted in the anti-feminist rhetoric. This identity is proudly flaunted as a marker of choice and superiority, even in conversations with unknown women online; one such comment was “I chose MGTOW because I prefer to keep all my life’s earnings, avoid the cheating practices of women, and avoid unnecessary stress and drama.” The core tenets of MGTOW are situated in the MRA discourse; this movement is characterized by the assertion that women hold unfair systemic and social advantages as a result of the feminist movement, which has “oppressed” men (O’Donnell, 2020). This assertion was seen in the comments like:

Men’s Rights Activism is for men that have dealt with the system up front and personal. It’s for men that have dealt with abusive sisters, mothers and girlfriends. It’s for men that have been chewed up and spit out by divorce courts and realize that marriage is not a good deal for men. It’s for men that are tired of the double standards in society that hurt men.

By saying this, MRAs adopt a defensible position as the suffering victim, turning feminist activism on its head and re-framing it as oppressive (Marwick and Caplan, 2018). MRA and MGTOW, which until recently were used almost exclusively within the manosphere, functions as part of a common linguistic practice on social media. This creates a sense of community across divergent subgroups, builds ties between individuals, and helps to solidify the ideological commitment of MRAs to oppose feminism. It also exists as a tool to counter feminist language and ideas (Marwick and Caplan, 2018).

The *Incels* group is closely associated with the MRA; they are self-identified “involuntary celibates” harboring hostility toward women for denying them sex, which they believe they inherently deserve (Jones et al., 2019). However, this group of the manosphere did not assert their identity, but it was rather used by women as a way of trolling men. Any instances of anti-feminist comments by men were countered or challenged by terming it as “Incel” with a discursive implication of ridiculing and dismissing the other, as seen in a comment: “Ignore him. It’s a faceless incel troll who posts here because this is the only way he will get any interaction with women. Otherwise rejected product in real life.” The creation of fake IDs, abusing women online, keeping their identity anonymous and getting blocked were some of the behaviors that female participants called out in their use of the *Incel* discourse for any man online, whether they actually identified as such or not: “No one as useless as faceless incel trolls here who made dozens of fake IDs to spam, abuse women daily and post illogical nonsensical comments even after their IDs are restricted repeatedly.” It is interesting to see how the word is used in interactions between ideologically competing groups, during which both MRAs and feminists negotiate the meaning of *Incel*. In such instances, each group defines and makes meaning of the word according to their own ideologies and beliefs (Marwick and Caplan, 2018). While Incel has a shared meaning, it is leveraged toward different ends. Thus, the use of the term is action- or -practice-oriented, serving to orient one group toward another: Incels against feminists, or feminists against Incels. Additionally, it is important to mention the emergence of the discourse on Manosphere in the Indian (and similar) context. MGTOW is not a familiar term in offline spaces yet, highlighting the influence of social media in creating and reframing gender discourses. These findings support claims made by the SIDE model, that minority influence and activism can help shift opinions toward itself in online settings (Perfumi, 2020).

We also noted the frequently occurring term *Mansplaining*. The origins of mansplaining can be traced back to a 2008 blog post titled “Men explain things to me” (Solnit, 2012). The term is generally used to refer to an explanation, usually offered by a man, which is patronizing, condescending, or ignores women’s experience and knowledge (Rothman, 2012). In our data, mansplaining was often used by women as a counter to assertions of the perceived manosphere: “Women here know better than trolls and need no mansplaining on any side of any story”; “You learn cooking yourself before mansplaining and lecturing women.” Using hashtags like #Mansplaining is a way to draw from dominant discourses on gender that heavily influence interactions online. It includes a performative aspect of social identity and what is believed to be prototypical in-group behavior (Postmes and Spears, 2013). This term is widely used and has become a common signifier of the feminist discourse (Lutzky and Lawson, 2019). Thus, women often used this term, irrespective of the comment by the outgroup, to uphold ingroup norms and assert their salient identity values that have become markers of the widely perceived feminist discourse, especially on social media.

### Intersectional Identities

Intersectionality or the interconnected nature of social categorizations such as race, class, and gender as they apply to a given individual or group is a core element of feminist analysis (Aldoory et al., 2008). In the Indian context, this intersectionality is particularly salient between four facets of one’s social identity—gender (male or female), caste (Upper and lower or Dalits), religion (Hindu or Muslim), and political ideology (Right wing or Left and Liberal). We noted several emerging themes that represent both the establishment of new identity norms (for feminism and gender identity) as well as challenge the power dynamics within existing narratives. Here, labels like *Savarna Liberal Feminism* and *Urban Feminism* were used to underline differences within this shared discourse. Speakers questioned the ideological intention of others by emphasizing their privilege and highlighting the elite nature of Indian feminism that has a liberal, usually urban and upper-caste or *Savarna* perspective. Comments like “Savarna liberal Feminism will never talk about a Dalit woman” and “Ever heard of Dalit Feminism? Please read more about it” point to this. Another term, Choice *Feminism* was used to indicate the selective and individualistic nature of feminism, with one participant commenting, “Fuck your liberal choice feminism. It’s completely toothless against the patriarchy, as you only think about yourself. You liberals and your individualistic policies.” Another comment read:

These urban feminists will not fight the real fires faced by women in serious oppressive conditions, but will create a pseudo crisis, where there is none, so that you scream fire and do the bare minimum without getting your hands dirty in the real mess.

Interestingly, men used intersectionality as part of the antifeminist discourse, where the ideology of feminism was coupled with right wing extremism, to delegitimize the claim. One of the comments was, “Are you really a feminist? You sound more like a fascist andh bhakt. Control your emotions and stop telling men what to do or not to do.” While the stereotype that feminists hate men is as old as feminism itself, adopting “facism” as a synonym for “feminism” allows men to appropriate the language of authoritarian identity politics and claim a victimized stance. In contemporary India, the Sanskrit term *bhakt* is used to denote supporters of the Hindu right wing, to equate their following with a devotee’s blind faith in their deity (Khan, 2015). In this discursive exchange, we note a strategic identity performance where a separate but intersectional identity is used to underline authoritarianism and extremism in outgroup’s stance, thereby demobilizing them (Klein et al., 2007). Interestingly, the *bhakt* or right-wing label is more popularly used for a masculine, militarized stance dominated by men (Grewal, 2020), but in these interactions, similar to the use of the term “Incel” we see the strategic use of a common term, in contrasting ways by men and women.

### Threat, Sex, and Violence

In the themes discussed so far, we found many argumentative, defensive, and critical interactions between the speakers. However, there were a few recurring terms that were particularly and intentionally offensive and violent. Most of these related to sexual habits and choices and were used when describing behaviors perceived as threatening to accepted societal norms. These included *#Slut* for women and *#Fuckboi* and *Simp* for men. As identified by Peters (2017), the term “fuckboy” (alternatively spelled “fuckboi” or “fuccboi”) is the first sexualized insult for men and most studies point to this character as a careless, misogynistic, and sex infatuated man with an absence of social skills. In our analysis, we saw the use of “#Fuckboi” by women when they were labeled as “sluts” or “whores” by men, through comments like, “Fuckbois act so fragile that even an article which has nothing to do with them hurts their glassy balls.” Several comments included terms against men who supported women in the comment threads, with the use of terms like “Sissy,” “Simp,” and “Small Cock.” The term *simp* refers to a male who overly desires female attention (Lomas, 2018-2019), thus seen as an outlier of the manosphere. One such comment was: “My anti feminism is not women hating. But I hate simps though.” In another instance, a male participant questioned the number of likes a comment by a woman had received, by calling out the men who had liked it: “The saddest part is a few of the likes she got for that comment is from simping small cocks.” As argued by Jones et al. (2019), the real tension for men is to prove their in-group membership by demonstrating a rejection of women. Such a rejection is more of a performance for their male peers, rather than a specific and deliberate attack on women. Demonstration of this masculinity also involves the rejection of non-masculine men. This corroborates findings that the presence of women on social media is seen as an ambitious threat to the notions of Indian masculinity (Halder and Jaishankar, 2016).

Using hateful and violent language or flaming has been a recurring area of enquiry in CMC research (Postmes et al., 2000; Moor, 2007; Hutchens et al., 2015). Identification with the ingroup and perceptions of offending by the outgroup, predicts why individuals flame in an online context. Beyond self-directed or individual factors, social identity plays an important role in online flaming. Our data support previous work, given the consistent presence of reactive aggression throughout the exchanges (Hutchens et al., 2015). Comments perceived as threatening to the ingroup social identity were met with particularly hostile responses (Moor, 2007; Perfumi, 2020). In the analyzed comments, where gender was salient due to the nature and themes of conversation as well as self-identification and categorization of speakers, the performance of identity was persistently aggressive, largely dismissive of the outgroup, as well as creative in its ability to use terms in ways that are self-serving to the in-group. When social identity cues are visible and relevant in an online context (as in these Facebook pages), participants are more likely to stereotype outgroup members. We noted the strong presence of gender stereotypes that ranged from traditional offline references to relatively novel terms indicating the emergence of new definitions of gender identity. Hutchens et al. (2015) found that online norms supporting flaming was an important determinant of flaming behavior and participants who used online platforms where political flaming was common, were more likely to do the same themselves. Most of our data included highly contested discussion on pages where flaming may be common. Thus, individuals interacting on these pages could perceive this as normative and use aggressive defenses more readily.

Due to the limited scope of our study, we were unable to examine a larger data corpus across a wider range of online pages, which may reveal differences in discursive content. Even though our data corpus picks on intersectionality, it does not completely reflect India’s masculinities and femininities and its rather large offline space. Moreover, even in the online space, future research must investigate a wider corpus of online gender discourses to confirm the consistency of these themes and potentially reveal more cross-cultural discourses. While this analysis sheds light on how the internet has ushered in a new era of digital activism and identity performance, it falls short of elucidating the long-term implications of such discursive digital identities. For instance, our findings are in line with previous research highlighting online incivility and its potential for polarizing discourses among politically aligned groups by highlighting similar patterns of uncivil discourses among gender groups (Anderson et al., 2018). This underscores the need to focus on group-based interactions on social media and its long-term implications beyond political affiliation, to other contested identities. Studying online movements like MRA and Feminism leaves significant gaps in our knowledge of the specific emotions and justification of the speakers. We invite researchers to look into these gaps in the hopes of shedding light on such complexities.

## Conclusion

We examined social media interactions between men and women on public Facebook pages, around the contested themes of feminism and gender. In doing so our main aim was to explore these discursive strategies as social identity performances that are goal directed and normative. We analyzed the data with reference to the context which is marked by transforming gender understandings, and identified the emergence of new forms of discursive activism in online forums. We found that speakers conflicted over the discourse of feminism in various ways, by using traditional as well as novel terms that refer to descriptive meanings of gender categories. These included new discourses within feminism (pseudo feminism and choice feminism) and men’s rights (Incels, MGTOW, etc.). There were also instances of flaming where the traditionally contested space of sex and sexual choice was used to challenge shifting gender roles. Lastly, interactions also highlighted several challenges to established meanings of feminism, by pointing toward intersectional identities. The findings add to the examination of digital influences on changing gender relations in the Global South, specifically from a social psychological perspective. They highlight how social identity and related norms are evolving through online interactions and shaping changing meanings and constructs of gender. As Perfumi (2020) suggests, these findings can contribute to an engaged understanding of normative influences on social media interactions and be particularly helpful in identifying both positive identity assertions by historically disadvantaged groups, as well as the negative consequences of online flaming and identity polarization. Moreover, the development of new discourses that are born out of digital spaces and interactions can extend beyond online communities to influence offline identification and gender relations. Thus, the findings reiterate a complicated and critical understanding of CMC that is both enabling gendered expressions and at the same time reinforcing gender-based anxieties that could result in unfulfilling and negative social media experiences.

## Data Availability Statement

The original contributions presented in the study are included in the article/supplementary material; further inquiries can be directed to the corresponding author.

## Ethics Statement

The studies involving human participants were reviewed and approved by Ashoka University Institutional Review Board. Written informed consent for participation was not required for this study in accordance with national legislation and institutional requirements.

## Author Contributions

SM was the principal investigator and responsible for designing and undertaking the study overall, specially analysis and reporting. MT was the lead researcher and has worked on the method and design as well as data collection. DJ and KB were research assistants involved in data collation and supervised analysis, literature review and writing. All authors contributed to the article and approved the submitted version.

## Conflict of Interest

The authors declare that the research was conducted in the absence of any commercial or financial relationships that could be construed as a potential conflict of interest.

## Publisher’s Note

All claims expressed in this article are solely those of the authors and do not necessarily represent those of their affiliated organizations, or those of the publisher, the editors and the reviewers. Any product that may be evaluated in this article, or claim that may be made by its manufacturer, is not guaranteed or endorsed by the publisher.
